# Flow-sensitive four-dimensional (4D) magnetic resonance imaging reveals abnormal blood flow pattern in the aorta and pulmonary trunk of patients with transposition of the great arteries operated with atrial baffle switch

**DOI:** 10.1186/1532-429X-14-S1-W67

**Published:** 2012-02-01

**Authors:** Sarah Nordmeyer, Eugenie Riesenkampff, Daniel Messroghli, Felix Berger, Titus Kuehne

**Affiliations:** 1 Department of Congenital Heart Disease/Pediatric Cardiology, Deutsches Herzzentrum Berlin, Berlin, Germany

## Background

Patients with transposition of the great arteries (TGA) after atrial baffle switch operation show differences in ventricular torsion and outflow tract geometry compared to healthy volunteers. We sought to investigate if these differences in cardio-mechanics translate into abnormal blood flow patterns in the pulmonary trunk and the aorta.

## Methods

Blood flow patterns were assessed with flow-sensitive four-dimensional velocity-encoded magnetic resonance imaging (4D VEC MRI), using a 1,5T Phillips MRI system. Measurements were made in the pulmonary trunk and the aorta in healthy volunteers (n=7) and TGA patients after atrial baffle switch operation (n=10). Blood flow was analyzed for vortex formation using custom-made software.

## Results

There were clear differences in blood flow patterns between healthy volunteers and TGA patients in both the pulmonary trunk and the aorta. In healthy volunteers, flow was laminar, parabolic in the pulmonary trunk and showed left helical flow pattern in the aorta. In TGA patients we observed opposite flow patterns with predominant parabolic flow in the aorta but helical flow and vortex formation in the pulmonary trunk.

## Conclusions

There are abnormal flow profiles in the aorta and pulmonary trunk in TGA patients. The data of this study provides evidence that differences in left and right ventricular cardio-mechanics directly translate into different flow patterns.

